# glmGamPoi: fitting Gamma-Poisson generalized linear models on single cell count data

**DOI:** 10.1093/bioinformatics/btaa1009

**Published:** 2020-12-09

**Authors:** Constantin Ahlmann-Eltze, Wolfgang Huber

**Affiliations:** Genome Biology Unit, EMBL, Heidelberg 69117, Germany; Genome Biology Unit, EMBL, Heidelberg 69117, Germany

## Abstract

**Motivation:**

The Gamma-Poisson distribution is a theoretically and empirically motivated model for the sampling variability of single cell RNA-sequencing counts and an essential building block for analysis approaches including differential expression analysis, principal component analysis and factor analysis. Existing implementations for inferring its parameters from data often struggle with the size of single cell datasets, which can comprise millions of cells; at the same time, they do not take full advantage of the fact that zero and other small numbers are frequent in the data. These limitations have hampered uptake of the model, leaving room for statistically inferior approaches such as logarithm(-like) transformation.

**Results:**

We present a new R package for fitting the Gamma-Poisson distribution to data with the characteristics of modern single cell datasets more quickly and more accurately than existing methods. The software can work with data on disk without having to load them into RAM simultaneously.

**Availabilityand implementation:**

The package glmGamPoi is available from Bioconductor for Windows, macOS and Linux, and source code is available on github.com/const-ae/glmGamPoi under a GPL-3 license. The scripts to reproduce the results of this paper are available on github.com/const-ae/glmGamPoi-Paper.

**Supplementary information:**

Supplementary data are available at *Bioinformatics* online.

The statistical distribution of sequencing counts from single-cell RNA-Seq can be modeled by y∼GammaPoisson(μ,θ) (Grün *et al.*, 2014; Hafemeister and Satija, 2019; Silverman *et al.*, 2018; Svensson, 2020), where *y* are the observed counts for a particular gene across a set of sufficiently similar cells (acting as replicates) and *μ* represents the underlying, true expression level of the gene (the expectation value). The parameter θ≥0 determines the dispersion of the distribution; the tightest case is *θ *= 0, in which case the distribution coincides with the Poisson distribution. Larger values of *θ* correspond to wider distributions.

Biological interest is added by extending the model beyond (conceptual) replicates and letting *μ* vary across the cells. This can be done in different ways: via a generalized linear model, log μ=Xβ, as in the differential expression methods edgeR (McCarthy *et al.*, 2012; Robinson *et al.*, 2010) and DESeq (Anders and Huber, 2010; Love *et al.*, 2014); via a factor analysis model (Risso *et al.*, 2018); or via a matrix decomposition analogous to PCA (Townes *et al.*, 2019). The model fits then provide biological insight about ‘significant’ variations in gene expression across cells, above and beyond the sampling noise.

A popular alternative approach is to transform the counts using the shifted logarithm f(x)=log(x+c), with some choice of *c *>* *0, and then proceed with analysis methods that are based on the least squares error, such as used for normal distributed data. However, this approach is fundamentally inferior as it overemphasizes the influence of small count fluctuations (Silverman *et al.*, 2018; Warton, 2018) and deals poorly with variable sequencing depth across cells (Townes *et al.*, 2019).

With the Gamma-Poisson generalized linear model, parameter estimation proceeds by minimizing the deviance, a generalization of the sum of squares of residuals used in the least squares method. There are already a number of implementations to this end, including the R packages MASS (Venables and Ripley, 2002), edgeR and DESeq2. These all follow a similar approach: for each gene, the parameter vector *β* is estimated using an iterative reweighted least squares algorithm, and the dispersion *θ* is found by likelihood maximization. After years of development, and with tens of thousands of users, edgeR and DESeq2 provide robust implementations and are a *de facto* standard for the analysis of bulk RNA-seq data Application of these implementations to single-cell RNA-seq data, however, suffers from several issues. First, their runtime becomes excessive as the number of cells gets large. Second, their functionality—fitting a Gamma-Poisson generalized linear model for a fixed, known design matrix *X*—is only part of what users need: with single-cell RNA-seq data, important research questions include identification of latent factors, dimension reduction, clustering and classification. These limitations hamper the development and uptake of statistical models based on the Gamma-Poisson distribution and appear to be driving analysts toward the transformation approach.

The R package glmGamPoi provides inference of Gamma-Poisson generalized linear models (details of the algorithm in Supplementary Appendix S1) with the following improvements over edgeR and DESeq2:

1. Substantially higher speed of the overdispersion estimation, by using an efficient data representation that makes uses of the fact that most entries in the count matrix are from a small set of integer numbers ({0,1,2,…}).
2. Better estimates (i.e. larger likelihood) of the overdispersion on datasets with many small counts.
3. No size limitations for the datasets. glmGamPoi supports fitting the model without loading all data into RAM simultaneously (i.e. working ‘on-disk’), by using the HDF5Array (Pagès, 2020) and beachmat (Lun et al., 2018) packages.
4. Small number of R package dependencies to facilitate use as a building-block for higher-level methods, such as factor analysis, dimension reduction or clustering/classification.

Like edgeR, glmGamPoi also provides a quasi-likelihood ratio test with empirical Bayesian shrinkage to identify differentially expressed genes (Lund et al., 2012). In addition, it provides the option to form a *pseudobulk* sample, which Crowell et al. (2019) found to be an effective way to identify differential expression between samples for replicated single cell experiments.

To demonstrate how glmGamPoi can be integrated into other tools, we forked the DESeq2 package and integrated glmGamPoi as an alternative inference engine (github.com/mikelove/DESeq2/pull/24).

We compared the runtime of glmGamPoi to other methods on the four single cell datasets summarized in Supplementary Table S1. The timing results are shown in Figure 1 and Supplementary Figure S1. The speedup by glmGamPoi compared to edgeR and DESeq2 was 6× to 13×. When the data were accessed directly from disk, the calculations took about twice as long. Omitting the estimation of *θ* (by setting it to zero) reduced the runtime to about a half. The forked version of DESeq2 that calls glmGamPoi was about as fast as calling glmGamPoi directly, indicating that inference carried out by glmGamPoi uses the largest part of the compute resources, while the additional steps carried out by DESeq2 make relatively small demands. Although all methods theoretically scale linearly with the number of genes and cells, we find empirically that glmGamPoi scales sub-linearly with the number of cells, which explains the observed performance benefit (Supplementary Fig. S2).

**Fig. 1.**
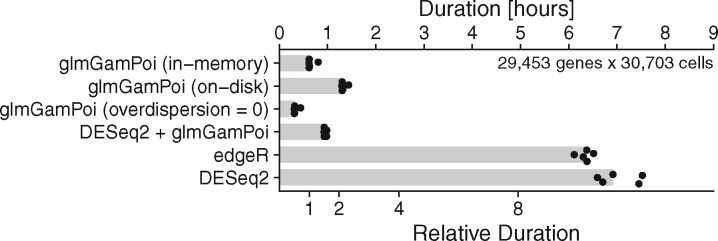
Bar plot comparing the runtime of glmGamPoi (in-memory, on-disk and without overdispersion estimation), edgeR and DESeq2 (with its own implementation, or calling glmGamPoi) on the Mouse Gastrulation dataset. The time measurements were repeated five times each as a single process without parallelization on a different node of a multi-node computing cluster with minor amounts of competing tasks. The points show individual measurements, the bars their median. To reproduce the results, see Supplementary Appendix S2

On the PBMC68k dataset, the calculations of DESeq2 and edgeR aborted because they ran out of memory (250 GB of RAM available). In contrast, glmGamPoi completed after ca. 45 min (Supplementary Fig. S1).

Supplementary Figures S4–S6 show that glmGamPoi’s gain in performance does not come at a cost of accuracy. On the contrary, Supplement Figure S3 shows that glmGamPoi provides better estimates (in the sense of larger likelihood) than DESeq2 for 72% of the genes and 10% of the genes in comparison with edgeR. Those differences with edgeR, seem to be of minor importance for assessing differential expression: the bottom rows of Supplementary Figures S5 and S6 show that the *P*-values from glmGamPoi and edgeR are very similar, consistent with the fact that they use the same statistical test. In Supplementary Figure S7, we provide a more detailed comparison for which genes the *P*-values of glmGamPoi and DESeq2 are similar and for which genes they are different.

## Funding

This work was supported by the EMBL International PhD Programme. In addition, this work has received funding from the European Research Council Synergy Grant DECODE under grant agreement No. 810296.

*Conflict of Interest*: none declared.

## Supplementary Material

btaa1009_Supplementary_Data
