# Supplementary material for: glmGamPoi: fitting Gamma-Poisson generalized linear models on single cell count data
Source: Bioinformatics. 2020 Dec 9;36(24):5701–2. doi: 10.1093/bioinformatics/btaa1009 (PMC8023675; doi:10.1093/bioinformatics/btaa1009)
Supplement: btaa1009_Supplementary_Data [file btaa1009_supplementary_data.pdf]

1 Methods

This section provides an overview over the implementation of `glm_gp`, the main function of the package `glmGamPoi`. `glm_gp` takes as input a count matrix and a specification of the design formula. In addition, it has several flags to turn on or off individual steps: size factor estimation, overdispersion estimation, overdispersion shrinkage, Cox-Reid adjustment. After validating its input, the function goes through the following steps and finally returns an estimate for the overdispersion for each gene, the regression coefficients for each gene and covariate, the deviance, the mean matrix and the size factors.

1. Estimate the size factors. `glmGamPoi` offers three different estimation techniques:
   - Column mean
   - Median of the ratios for each sample compared with a pseudo-reference sample (described by equation 5 in Anders and Huber (2010)). To avoid issues with the zeros, we calculate the ratio only on the positive counts.
   - A deconvolution based method calling `scran`'s `calculateSumFactors` function

   The tuple of all size factor estimates is scaled by a common factor such that their geometric mean is one.

2. Estimate the overdispersion roughly using the empirical variances and the mean-variance relation of the Gamma-Poisson distribution $\sigma^2 = \mu + \theta\mu^2$.

3. Check if the experimental design is a single factor variable, in which case the coefficients of the GLM can be estimated for each group individually (Case 1). Otherwise, Case 2 applies.

4. Estimate coefficients for each gene roughly.
   - Case 1: the estimates are the average counts per group.
   - Case 2: fit the linear model.

5. Estimate the coefficients for each gene properly.
   - Case 1: use a Newton-Raphson procedure to find the optimal coefficient for each group.
   - Case 2: use Fisher-Scoring with a small (10^{-6}) ridge penalty in combination with a line search to make sure each step decreases the deviance to find the coefficients for each gene.

6. Calculate the mean matrix $M = \exp\left(BX^T + D\right)$, where B is the coefficient matrix, X is the design matrix, and D is the offset matrix.

7. Estimate the overdispersion for each gene properly by optimizing the log-likelihood of the Gamma-Poisson GLM. The optimization calls the `nlminb` function in `R` and uses the analytical expression of the first and second derivative to guide the optimization.
   - If the first derivative is negative for $\theta \leq 10^{-8}$, we assume that there is no maximum and set the overdispersion estimate to zero
   - We create a frequency table $\text{F} = \{\{0, f_0\}, \{1, f_1\}, \{2, f_2\}, \ldots\}$ for each count vector $\boldsymbol{y}$, where f_k is the number of times that the count value k occures for that gene. We use this table to speed up parts of the log-likelihood calculation. For example, instead of calculating the log-gamma function for each element

     $$r = \sum_{i=1}^{n} \log\Gamma(y_i),$$

     where $n = \text{length}(\boldsymbol{y})$, we calculate

     $$r = \sum_{(k,f_k)\in F} f_k \log\Gamma(k).$$

     This optimization contributes most to the speed-up, because the table F is much smaller for many genes than the full vector $\boldsymbol{y}$. If we realize during the creation of the frequency table, that it grows too large and the additional cost of creating the table is not worth it, we revert back to the standard vector based optimization.
   - The calculation of the first derivative is made more robust against numerical divergences by adding bounds on two terms of the sum.

– For small θ, the term $r = 1/\theta \left(\sum_{i=1}^{n} \psi(y_i + 1/\theta) - n\psi(1/\theta)\right)$ can become too large. We introduce an upper bound based on the Laurent series expansion of the equation at $1/\theta = \infty$ which is $r_{\text{upper}} = \sum_i y_i - \left(\sum_i (y_i - 1)y_i\right)\theta/2$.
– For small $\mu_i\theta$ the following term becomes imprecise $r = \log(1 + \mu_i\theta) - \frac{\mu_i\theta}{1+\mu_i\theta}$. We use a Taylor expansion of $\log(1 + \mu_i\theta)$ at $\mu_i\theta = 0$ to derive an upper bound $r_{\text{upper}} = (\mu\theta)^2\frac{1}{1+\mu\theta}$ and lower bound $r_{\text{lower}} = (\mu\theta)^2\left(\frac{1}{1+\mu\theta} - 1/2\right)$ to stabilize the calculation.

8. Shrink the overdispersion estimates using the quasi-likelihood framework $\sigma^2 = \theta_{\text{QL}}\left(\mu + \theta_{\text{trend}}\mu^2\right)$ that `edgeR` has developed (Lund *et al.*, 2012).
   - We fit a local median regression through the mean gene expression values and maximum likelihood overdispersion estimates θ_{ML} to identify the dispersion trend
   - We convert the maximum likelihood overdispersion estimates to quasi-likelihood overdispersion estimates θ_{QL} using the relation $\theta_{\text{QL}} = \frac{1+\mu\theta_{\text{ML}}}{1+\mu\theta_{\text{trend}}}$.
   - We estimate the parameters of the variance prior (the degrees of freedom df_0 and scale τ_0^2 of an inverse Chi-squared distribution) using a maximum likelihood procedure.
   - We shrink the quasi-likelihood overdispersion estimates by taking the weighted mean between the trended prior and the quasi-likelihood overdispersion estimates

$$\theta_{\text{SQL}} = \frac{\text{df}_0\tau_0^2 + \text{df}\theta_{\text{QL}}}{\text{df}_0 + \text{df}}$$

   where θ_{SQL} is the shrunken quasi-likelihood overdispersion estimate.

9. Re-estimate the coefficients based on the updated overdispersion estimates.
10. Re-calculate the mean matrix based on the updated coefficients.

Furthermore, `glmGamPoi` provides functionality to do differential expression analysis with the `test_de` function. The procedure is based on the quasi-likelihood ratio test framework developed for RNA-sequencing data by Lund *et al.* (2012). The `test_de` function takes as input a reduced design which is used as the null hypothesis. We calculate the likelihood ratio (LR) as the difference of the deviance of the full model (d_{full}) and the deviance of the reduced model (d_{reduced}) for each gene

$$\text{LR} = d_{\text{reduced}} - d_{\text{full}}.$$

From the likelihood ratio, we calculate the F statistic

$$F = \frac{\text{LR}}{\text{df}_{\text{test}}\theta_{\text{SQL}}},$$

where df_{test} is the difference between the number of covariates in the full vs. the reduced model and θ_{SQL} is the shrunken quasi-likelihood dispersion estimate from Step 8. Finally, to calculate the p-values, we compare F against a F-distribution with df_{test} and df_{fit} degrees of freedom, where $\text{df}_{\text{fit}} = \text{df}_0 + \text{df}$ from Step 8. For convenience, the `test_de` function also calculates the multiple testing adjusted p-values using the Benjamini-Hochberg procedure (Benjamini and Hochberg, 1995).

2 Reproducibility

Software versions: R 3.6.2, `glmGamPoi` 1.1.5, `edgeR` 3.28.1, `DESeq2` 1.29.4. The scripts to reproduce the results of this paper are available on github.com/const-ae/glmGamPoi-Paper. The data underlying this article are available from Bioconductor at https://dx.doi.org/10.18129/B9.bioc.MouseGastrulationData, https://doi.org/doi:10.18129/B9.bioc.TENxBrainData, and https://doi.org/doi:10.18129/B9.bioc.TENxPBMCData.

References

Anders, S. and Huber, W. (2010). Differential expression analysis for sequence count data. *Genome Biology*.

Benjamini, Y. and Hochberg, Y. (1995). Controlling the false discovery rate: a practical and powerful approach to multiple testing. *Journal of the Royal statistical society: series B (Methodological)*, **57**(1), 289–300.

Lund, S. P., Nettleton, D., McCarthy, D. J., and Smyth, G. K. (2012). Detecting differential expression in RNA-sequence data using quasi-likelihood with shrunken dispersion estimates. *Statistical Applications in Genetics and Molecular Biology*, **11**(5).

3 Supplementary Table

ID	Description	Data Location (doi)	#cells
Mouse Gastrulation	Effects of a gene-knockout on mouse gastrulation	10.18129/B9.bioc.MouseGastrulationData	31,000
Brain20k	Mouse brain atlas	10.18129/B9.bioc.TENxBrainData	20,000
PBMC4k	Peripheral blood mononuclear cells	10.18129/B9.bioc.TENxPBMCData	4,000
PBMC68k	Peripheral blood mononuclear cells	10.18129/B9.bioc.TENxPBMCData	68,000

Suppl. Table S1: Single cell datasets. In each, the number of genes was approximately 30,000.

4 Supplementary Figures

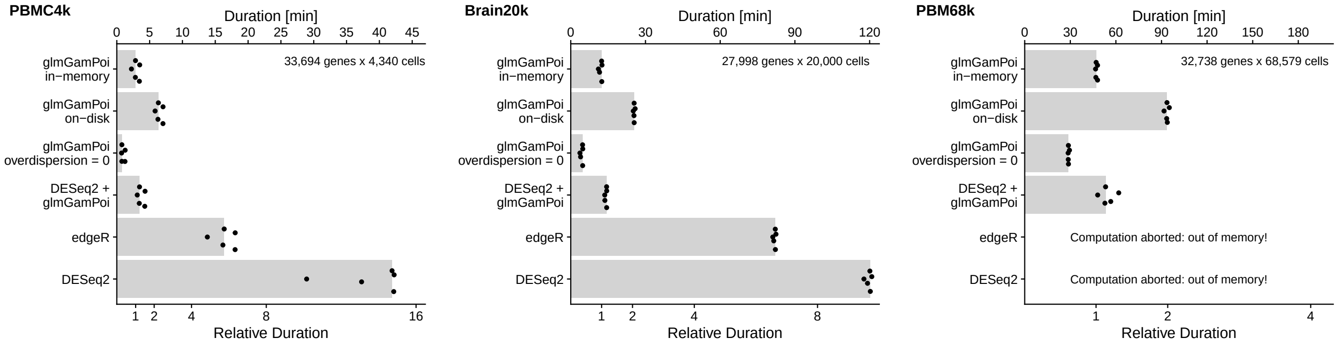

Suppl. Figure S1: Extension of Figure 1 with three additional datasets: PBMC4k, Brain20k, and PBMC68k. edgeR and DESeq2 aborted the computation on the PBMC68k dataset because the available memory of 250GB was not enough. The runtime on the Mouse Gastrulation dataset is larger compared with the other three datasets, because a more complex experimental design was fitted. It took into account the mutation status of the *tomato* gene, the developmental stage, and the sequencing pool, which resulted in 5 covariates in the design matrix. The experimental design for the Brain20k dataset accounted for the mouse that was sequenced (2 covariates). For the PBMC4k and PBMC68k, we used an intercept-model (1 covariate).

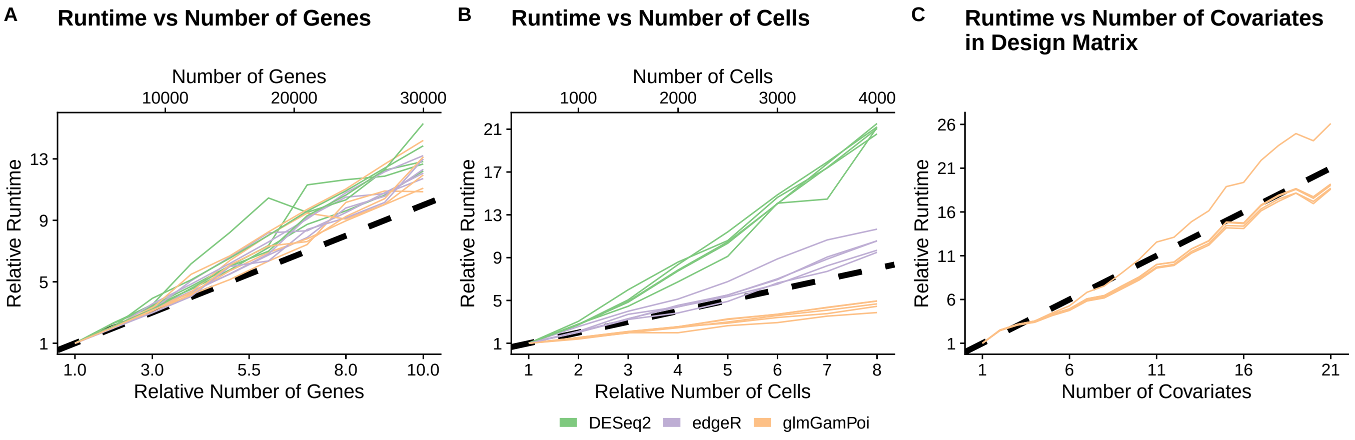

Suppl. Figure S2: Line plots that show the increasing runtime of `edgeR`, `DESeq2`, and `glmGamPoi` with A) the number of genes, B) the number of cells, and C) the number of covariates in the experimental design. The dashed black line marks the diagonal which implies linear increase of the runtime. The experiments were run on different subsets of the PBMC4k dataset. The same methodology for measurements as in Figure 1 was used. Panel C) shows only the runtime for `glmGamPoi` because the runtime for `edgeR` and `DESeq2` is dominated by the overdispersion estimation, which masks the effect of the increasing number of covariates. Although asymptotically the scaling with the number of covariates is cubic in `glmGamPoi`, because of the QR decomposition of the design matrix, for the limited number of covariates that are typically encountered in real-world experiments, the scaling is well approximated by a linear function.
The plot shows the relative durations, for reference we also provide the absolute runtime: In Panel A, a relative runtime of 1 corresponds to 12, 67, and 146 seconds for `glmGamPoi`, `edgeR`, and `DESeq2`, respectively. In Panel B, a relative runtime of 1 corresponds to 33, 75, and 88 seconds for `glmGamPoi`, `edgeR`, and `DESeq2`, respectively. In Panel C, a relative runtime of 1 corresponds to 19 seconds for `glmGamPoi`.

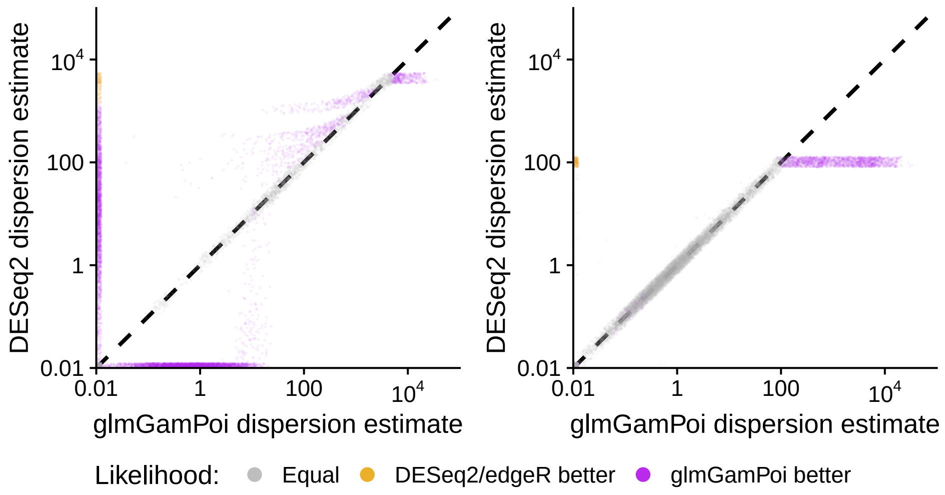

Suppl. Figure S3: Scatter plots on a log-log scale that compare the genewise dispersion estimates of `DESeq2` and `edgeR` against `glmGamPoi`. A small jitter is added to each point to avoid overplotting. All methods optimize the same Cox-Reid adjusted Gamma-Poisson profile likelihood. This means that the best algorithm given the same data is the one that achieves the largest likelihood. The grey points show genes for which the likelihood was approximately equal (i.e. within ±0.001). glmGamPoi achieved a larger likelihood for 14,675 / 1,958 (DESeq2 / edgeR) genes (purple), an equivalent likelihood for 4,698 / 17,385 genes (grey), and had a smaller likelihood for 400 / 430 genes (orange). The dashed lines mark the diagonal of the plot.

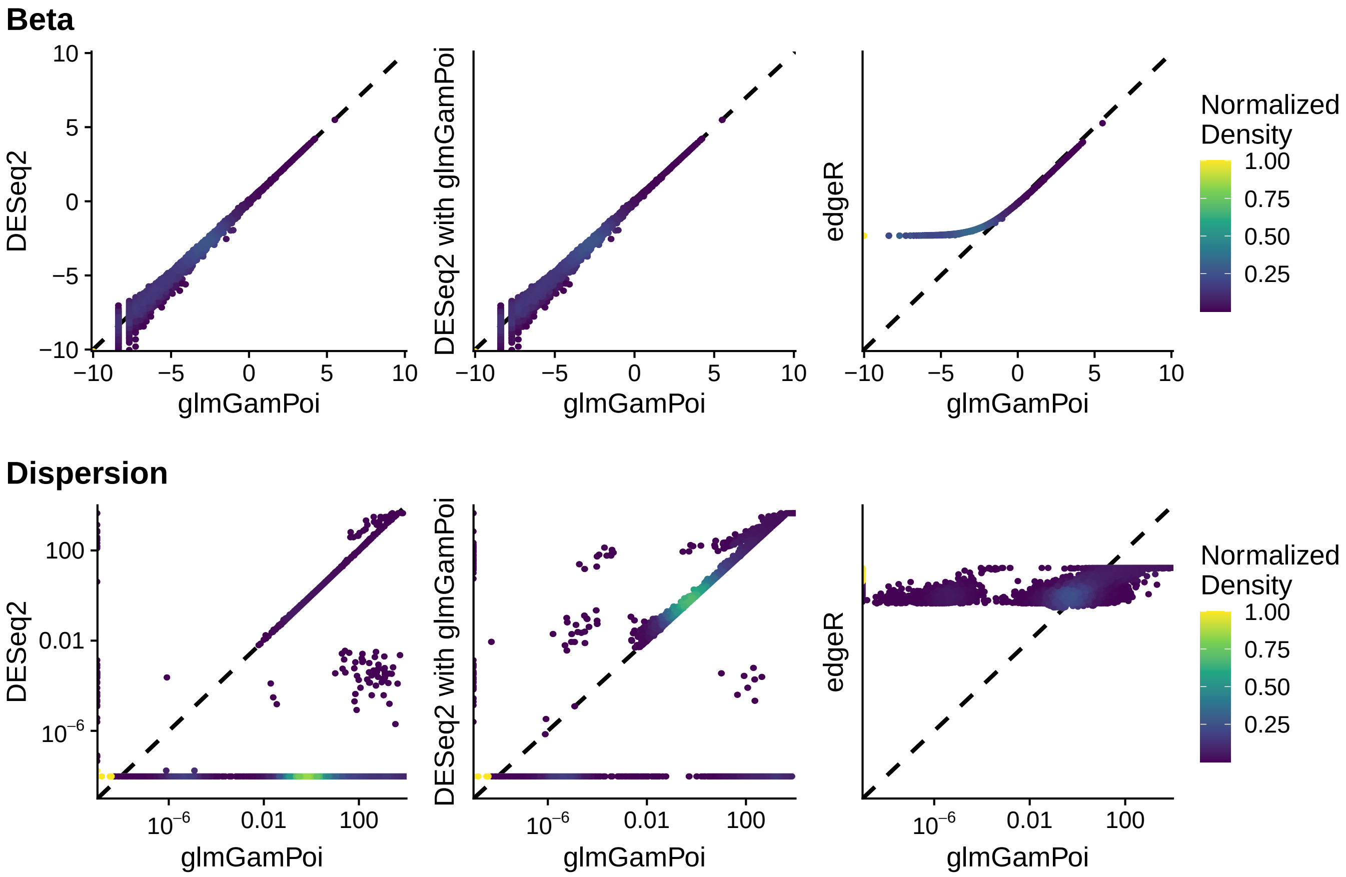

Suppl. Figure S4: Scatter plots that compare the estimates of the intercept (one column in the Beta matrix) and the dispersion on the PBMC4k dataset for DESeq2, DESeq2 calling glmGamPoi, and edgeR against glmGamPoi. The color of the points shows the density of points in the area. The dashed diagonal line marks the diagonal where the two estimates are identical. The density for the color is normalized within each plot. Note that the dispersion estimates of `edgeR` are the shrunken gene-wise estimates. This is because `edgeR` does not provide a straightforward way to extract the maximum likelihood estimates.

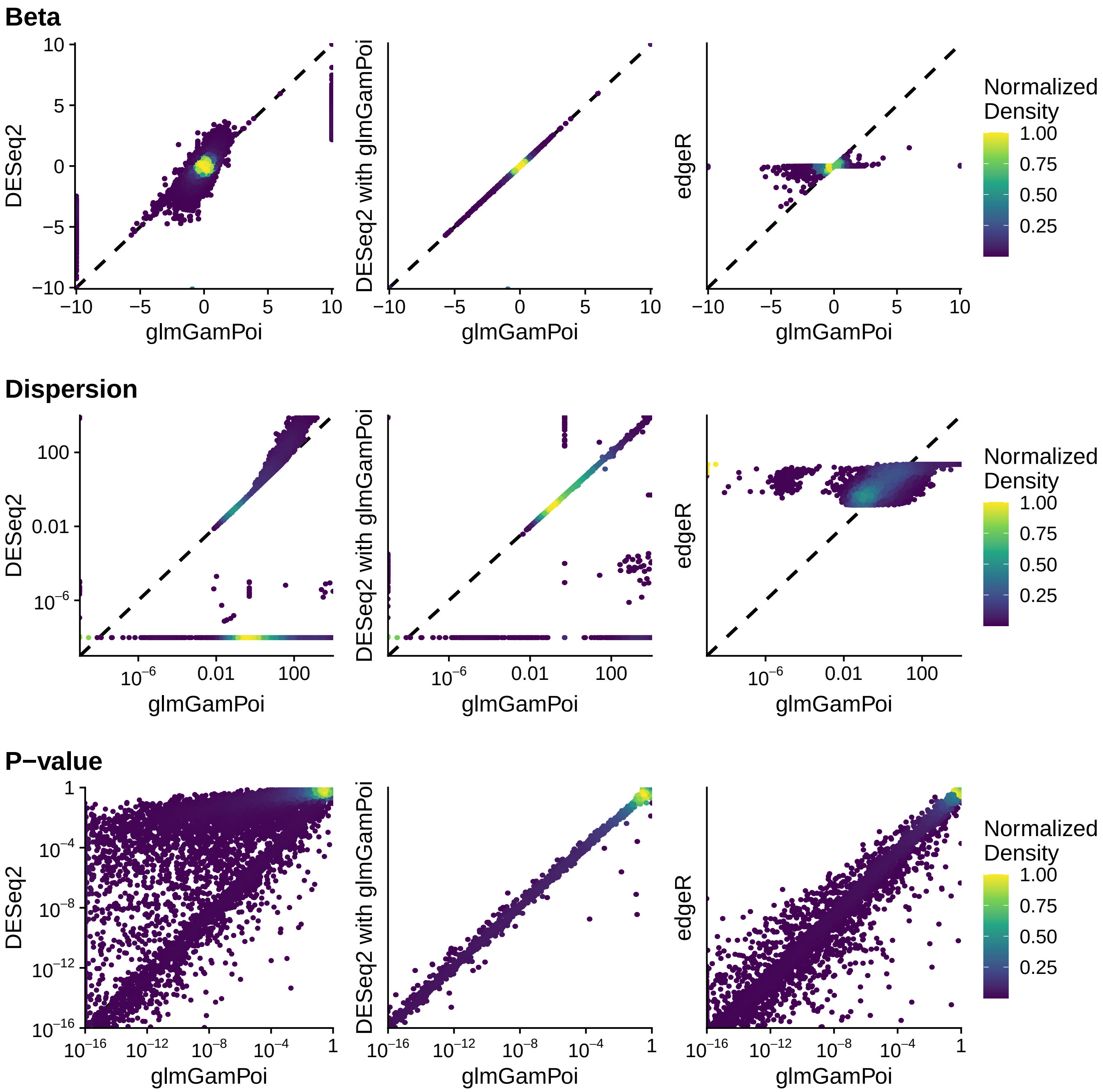

Suppl. Figure S5: Scatter plots that compare the estimates of the *tomato* coefficient (one column in the Beta matrix), the dispersion, and the p-value from the test for differential expression on the Mouse Gastrulation dataset for DESeq2, DESeq2 calling glmGamPoi, and edgeR against glmGamPoi. Otherwise it has the same structure as Supplementary Figure S4.

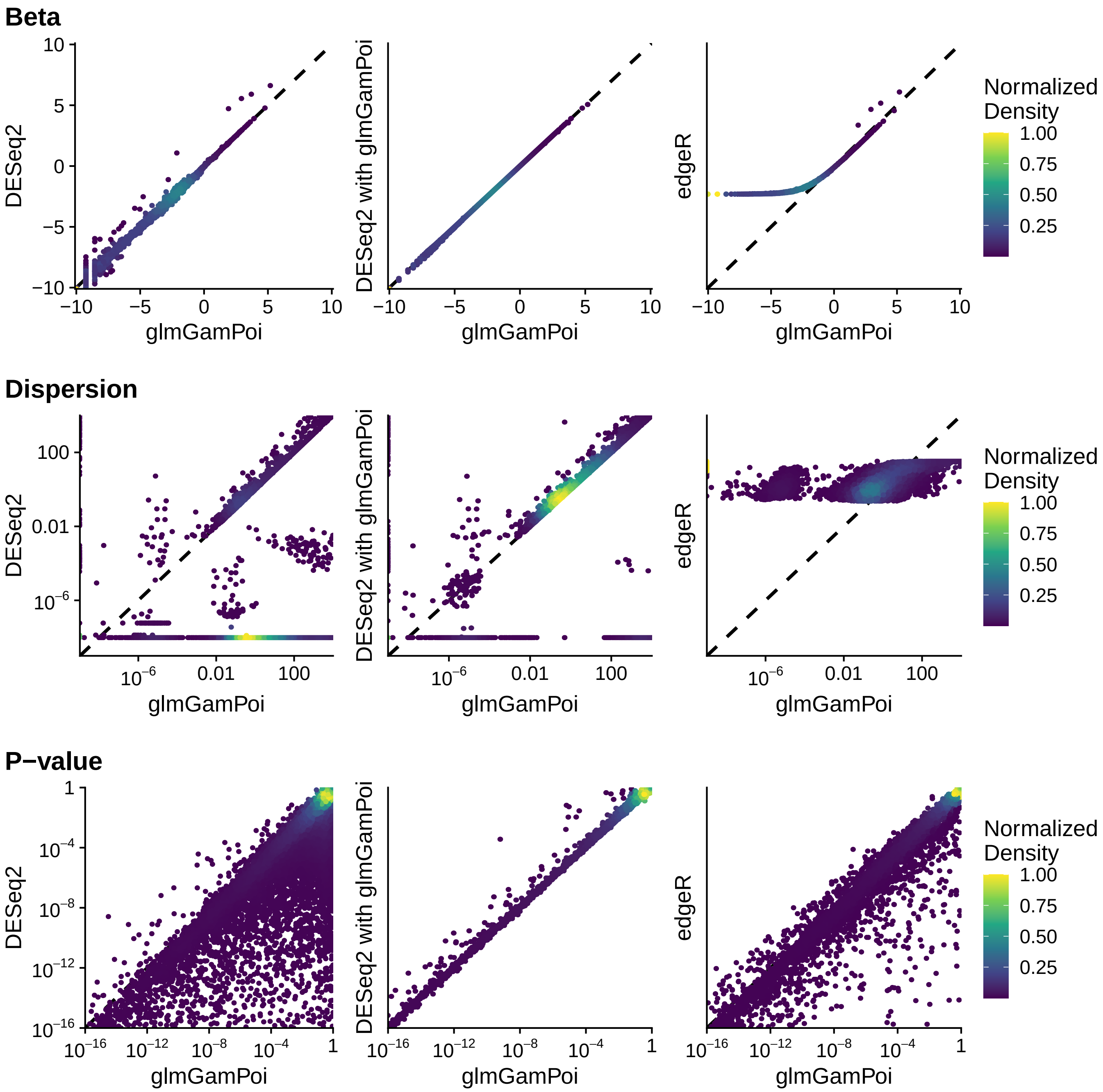

Suppl. Figure S6: Scatter plots that compare the estimates of the *MouseA* coefficient (one column in the Beta matrix), the dispersion, and the p-value from the test for differential expression on the Brain20k dataset for DESeq2, DESeq2 calling glmGamPoi, and edgeR against glmGamPoi. Otherwise it has the same structure as Supplementary Figure S4.

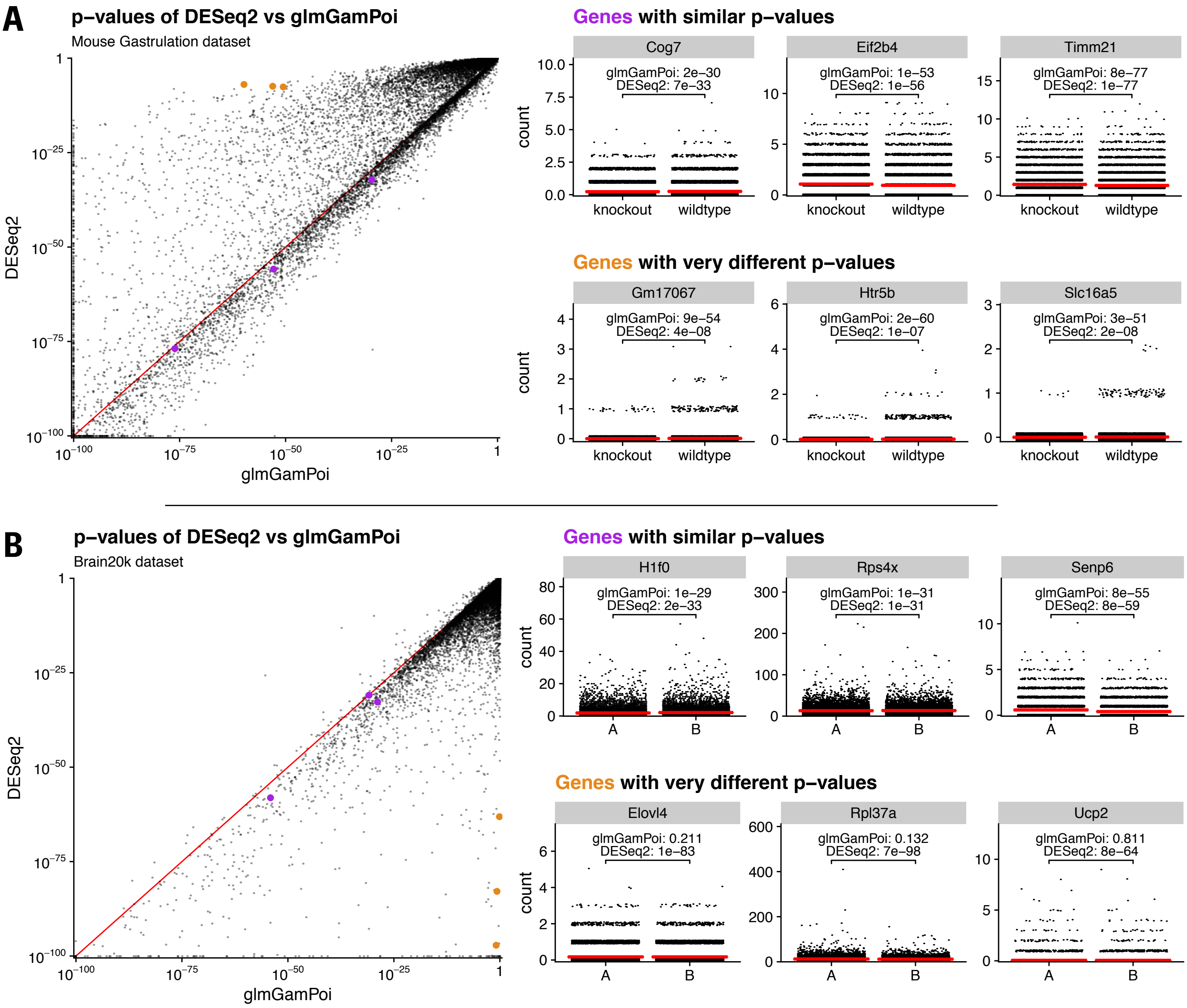

Suppl. Figure S7: Comparison of the p-values calculated with the likelihood ratio test of DESeq2 and the quasi-likelihood ratio test of glmGamPoi. A) shows the differences on the mouse gastrulation knockout dataset. B) shows the difference on the Brain20k dataset. The scatter plots on the left show the p-values for the full vs. the reduced design on a double-logarithmic scale. Three exemplary genes with similar p-value are highlighted in purple and three genes with very different p-values in orange. Their counts are shown on the right. The red lines show per-group mean.
